# Management of pregnancy in a patient with long‐chain 3‐hydroxyacyl CoA dehydrogenase deficiency

**DOI:** 10.1002/jmd2.12284

**Published:** 2022-04-12

**Authors:** Loai A. Shakerdi, Jenny McNulty, Barbara Gillman, Claire M. McCarthy, Jessica Ivory, Alison Sheerin, James J. O'Byrne, Jennifer C. Donnelly, Eileen P. Treacy

**Affiliations:** ^1^ National Centre for Inherited Metabolic Disorders Mater Misericordiae University Hospital Dublin Ireland; ^2^ National Centre for Inherited Metabolic Disorders Children's Health Ireland (CHI) Dublin Ireland; ^3^ The Rotunda Hospital Dublin Ireland

**Keywords:** acylcarnitine, long‐chain 3‐hydroxyacyl CoA dehydrogenase deficiency, pregnancy, rhabdomyolysis

## Abstract

Long‐chain 3‐hydroxyacyl‐CoA dehydrogenase deficiency (LCHADD) is a rare mitochondrial defect of β‐oxidation of long‐chain fatty acids. Patients may present with muscle pain, hypotonia, peripheral neuropathy, cardiomyopathy, recurrent rhabdomyolysis and sudden death. Dietary management of LCHADD aims at preventing prolonged fasting and decreasing energy production from long‐chain fatty acids compensated by an increase in medium‐chain triglyceride fat. Herein, we present medical and dietetic management of a successful pregnancy in a LCHADD female patient and the delivery of a healthy baby boy.

**SYNOPSIS**

Medical and dietetic management of a successful pregnancy in a patient with long‐chain 3‐hydroxyacyl‐CoA dehydrogenase deficiency.

## 1 INTRODUCTION

Long‐chain 3‐hydroxyacyl‐CoA dehydrogenase deficiency (OMIM 600890) is a mitochondrial defect of β‐oxidation of long‐chain fatty acids caused by mutations in the alpha subunit of the hydroxy acyl‐CoA dehydrogenase (*HADHA*) gene.

Defects of mitochondrial fatty acid metabolism may result in severe bioenergetic imbalance. Patients may present with muscle pain, hypotonia, peripheral neuropathy and cardiomyopathy.^1^ Acute metabolic decompensation may develop in conditions associated with increased energy demand such as fasting, severe illness or infection.

Creatine kinase (CK) and transaminases can be used as a parameter for monitoring treatment of long‐chain 3‐hydroxyacyl‐CoA dehydrogenase deficiency (LCHADD) patients.^2^, ^3^, ^4^ Elevated blood CK levels may be used to monitor rhabdomyolysis during metabolic derangement.^5^ CK levels >5000 IU/L are closely related to the development of kidney damage.^6^

Dietary management of LCHADD aims to prevent prolonged fasting by including a regular intake of carbohydrate and to decrease long‐chain triglyceride (LCT) to less than 10% of total energy whilst ensuring adequate intake of essential fatty acids (EFAs), specifically docosahexaenoic acid (DHA).^7^ Supplementation with medium‐chain triglycerides (MCTs) is recommended at 10%–20% of energy to optimise metabolic status by reducing the accumulation of toxic long‐chain 3‐hydroxylacylcarnitines.^7^ Age‐appropriate protein intake has been routinely suggested. However, recent publications have shown the benefit of increased energy intake from protein at 10%–25% of energy content. Adequate multi‐vitamins, fat‐soluble vitamins and minerals are required to supplement the diet.^7^, ^8^

Alterations in maternal lipid metabolism during pregnancy can be divided into an anabolic phase, occurring in the first two trimesters, and a catabolic phase.^9^ Accelerated breakdown of fat occurs during the third trimester.^10^

Reported complications in pregnancies in women with very‐long‐chain acyl‐CoA dehydrogenase deficiency (VLCAD), another genetic disorder of mitochondrial fatty acid β‐oxidation, include increased CK levels with myalgia and rhabdomyolysis.^11^ There is no specific guidance for the management of pregnancy in LCHADD. However, a recent publication by Van Calcar et al. recommends minimising periods of fasting, restrictions of LCT intake, MCT, l‐carnitine supplementation and provision of additional energy from carbohydrate and protein sources in the second and third trimesters of pregnancy with VLCAD.^12^ The first published report on the management of pregnancy in an LCHADD patient was in 2017.^13^

Herein, we present the medical, dietetic and maternal management of pregnancy and delivery of a healthy baby boy in a patient with LCHADD.

## 2 CASE DESCRIPTION

This case describes a 30‐year‐old female with known LCHADD. The patient has a complex medical background starting from 5 weeks old, when she began rapidly losing weight and eventually losing consciousness. LCHADD was diagnosed at 6 weeks of age following metabolic testing (urine organic acid analysis blood acylcarnitine profile (ACP) and fatty acid oxidation flux studies (Table S2). Genetic analysis subsequently confirmed homozygosity for the G1528C LCHADD disease causing variant in the alpha enzyme subunit of the HADHA protein. G1528C is a common LCHADD disease associated missense mutation.^14^

**FIGURE 1:**
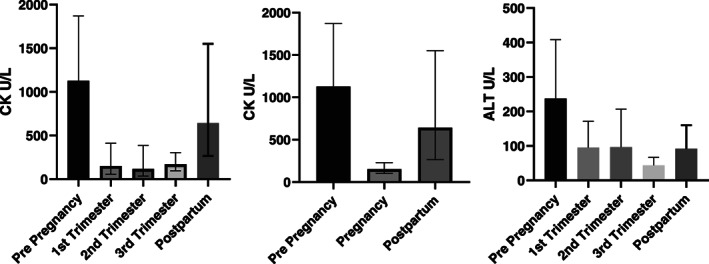
CK (U/L) and ALT (U/L) levels before pregnancy, at different stages of pregnancy and post‐partum (mean ± SD). ALT, alanine aminotransferase; CK, creatine kinase

The patient had recurrent episodes of acute metabolic decompensations at times of intercurrent infection (with presentations of hypotonia, hypoglycaemia and respiratory difficulties requiring recurrent ventilatory support). Subsequently, the patient has exhibited a number of long‐term complications secondary to LCHADD, including sensorimotor axonal neuropathy, bilateral pigmentary retinopathy, Grade III cardiac diastolic dysfunction and recurrent bouts of rhabdomyolysis. Our patient had four acute hospital admissions in the 4 years preceding her pregnancy with elevated CK levels (Figure 1). The highest CK level was reported at 45303 U/L 4 years ago. This elevation in CK was accompanied by urine discoloration and muscle symptoms.

She currently uses a crutch for short distance walking and a wheelchair for long‐distance travel due to significant peripheral neuropathy.

A pre‐conceptual discussion comprehensively addressed the risks of pregnancy and potential serious complications during the antenatal, peripartum and post‐partum periods. Within the 2 years prior to conception, fasting times were 4 h for daytime and 8 h for night‐time. She was adherent with the internationally recognised guidelines for the management of LCHADD, with restriction of LCT to less than 10% of total energy. She also included MCT supplementation to ~15% of total energy.^2^ She was routinely supplemented with DHA at 200 mg/day, a multivitamin and mineral supplement as well as a specific fat‐soluble vitamin every other day and carnitine 500 mg four times a day. She also took a modified corn starch supplement to support her overnight fast. She had an emergency management plan for use during periods of illness. The medications taken by the patient prior to pregnancy were pregabalin and topiramate, which were discontinued on confirmation of pregnancy, in addition to high‐dose folic acid and iron supplementation. A nerve conduction study was performed before pregnancy. It found evidence suggestive of a significant large fibre axonal neuropathy. No physiotherapy or occupational therapy was warranted at this stage. Our patient attends annual retinal screening. A preconception review was reported as being stable. Our patient attends annual cardiac assessment. The frequency of cardiac monitoring during pregnancy was guided by the attending cardiologist.

## 3 MEDICAL AND DIETETIC MANAGEMENT OF THE PREGNANCY

### 3.1 Trimester 1

The patient notified her metabolic team immediately on confirming the pregnancy, which was a spontaneous conception, and medical management was coordinated by a multi‐disciplinary team (MDT). Full cardiac and hepatic assessments were performed in the first and third trimesters. Echocardiography demonstrated an ejection fraction of 61% and borderline mild concentric hypertrophy. Her blood pressure at her initial visit was 129/80 mmHg. A hepatic ultrasound showed mildly increased echogenicity but no focal lesions. No visual changes were reported throughout the pregnancy.

An interventional radiology guided peripherally inserted central catheter was inserted at 9 weeks gestation due to poor venous access for regular phlebotomy. This also allowed for the possible infusion of hypertonic solutions such as glucose 15% or 20%. Full clinical and biochemical assessment was commenced at 5 weeks gestation and weekly thereafter. Biochemical investigations included CK, aspartate aminotransferase (AST), lactate dehydrogenase (LDH), urate, liver and renal profiles and full blood count (FBC). A transient rise in CK level was seen following influenza vaccination, which returned to normal without any intervention. Iron studies, bone profile, vitamin B12 and folate were monitored in accordance with the suggested pregnancy monitoring guidelines for VLCAD.^12^ ACPs, non‐esterified free fatty acids, EFA profile, fat‐soluble vitamins were monitored closely throughout the pregnancy as indicated. Levels of C18: 1‐OH‐acylcarnitine, C18‐OH‐acylcarnitine, C20:0 arachidic acid and the ratios of C22:5w3 docosapentaenoic acid and C20:3W6 homogamma–linolenic acid were increased, which is consistent with LCHADD profile. Acetylsalicylic acid supplementations were initiated at this stage. Our patient did not experience significant nausea or vomiting that required treatment.

Dietary management was adjusted based on her fortnightly weight, blood nutritional markers and liver enzymes. She was also reviewed by the obstetric team regularly to assess foetal wellbeing. LCT was restricted to ~9.5% of total energy, while protein intake was ~13% of total energy. The dietary prescription was altered to ensure vitamin and mineral intake was sufficient for pregnancy. This involved increasing DHA and EPA intake via an EFA acid supplement. She also remained on her fat‐soluble vitamin supplement, taking one tablet on alternate days.

### 3.2 Trimester 2

Dietetic review, biochemical investigations and blood pressure continued to be monitored on a weekly basis, along with patient weight, with the aim of 0.5 kg per week expected weight gain throughout the second trimester. Actual weight gain for the period was 0.56 kg per week. Estimated energy requirements were increased to reflect the second trimester requirement. Protein intake from diet and skimmed milk powder was increased to meet the dietary aim of ~15% total energy. MCT supplementation was also increased to achieve 20% total energy, using Betaquick (Vitaflo) and Monogen (Nutricia). It has been reported that MCT supplements could have positive impact on patient exercise tolerance and cardiac function.^15^ Intermittent elevations in CK levels were managed with additional intake of a glucose polymer product, SOS 25 (Vitaflo).

Erythrocyte EFA profile showed deficiency in linoleic acid and eicosapentaenoic acid (EPA) (Table S3). This was treated with the reintroduction of walnut oil as part of the LCT allowance of 10% total energy. Her EPA dose had already been increased in the previous 6 weeks period and therefore was not adjusted further. Low fat‐soluble vitamin levels were observed which resulted in an increase in the fat‐soluble vitamin supplement. The emergency dietary management plan was updated to reflect these changes. The local maternity hospital was also briefed on these changes. The patient's blood pressure was well controlled throughout the pregnancy.

From an obstetric perspective, serial growth ultrasounds were performed throughout the second and third trimesters. A foetal anatomy scan was carried out at 21 weeks gestation and did not detect any abnormalities, with the estimated foetal weight on the 75th centile. Following MDT discussion, it was decided not to vaccinate antenatally against pertussis owing to the previous CK rise following intramuscular influenza vaccination. Antenatal and breastfeeding education was provided by midwifery colleagues, as there is no known contra‐indication to breastfeeding.

### 3.3 Trimester 3

In anticipation of increased metabolic demands and risk of decompensation, the frequency of monitoring was increased. The expanded MDT (including anaesthesiology, neonatology and midwifery), held a number of meetings for the birth planning. Prophylactic low‐molecular heparin was prescribed. Twice weekly biochemical investigations were performed between the 30th week of pregnancy and the birth. A hepatologist was consulted and further tests were performed as indicated, including FBC, CK, LFTs, AST, LDH, prothrombin time, renal profile, urate, and urinary protein creatinine ratio. A number of acylcarnitine derivatives were found at their lowest levels during the third trimester (Table S4).

A detailed dietary analysis revealed adequate caloric intake for stage of pregnancy. Fat‐soluble vitamins were rechecked and her supplementation was reduced to one dose 5 days per week as her vitamin D level was above the normal threshold. Her vitamin A level remained persistently low; however, retinol binding protein levels were within normal limits. Haemodilution was cited as one of the explanations for the decrease in maternal concentrations of retinol during pregnancy.^16^ The patient's haemoglobin, ferritin and vitamin B12 levels began to trend downwards throughout the final trimester. She was prescribed additional oral vitamin B12 supplementation. An overview of the dietary prescription throughout pregnancy is shown in Table 1.

**TABLE 1:** Overview of dietary prescription throughout pregnancy

	% LCT fat	% MCT fat	% Protein	% CHO
Pre‐pregnancy	10.5	18.8	12	58.7
Trimester 1	9.5	15.8	13	61.7
Trimester 2	8.4	17.7	17	56.9
Trimester 3	9.6	19	16.5	54.9
Recommendations	10	10–20	15–20	45–50

Abbreviations: CHO, carbohydrate; LCT, long‐chain triglyceride; MCT, medium‐chain triglyceride.

A significant increase in CK (2954 U/L) in addition to moderate elevation in alanine aminotransferase (ALT) and AST was detected at 36 weeks gestation. There was no discolouration of urine or significant muscle symptoms reported.

The patient was thus admitted to the maternity hospital for monitoring, stabilisation of CK and preparation for the delivery. Antenatal corticosteroids were administered for foetal lung maturation intravenously in order to avoid a CK rise following deep intramuscular injection. An insulin sliding scale was introduced to control the associated hyperglycaemia.

The CK was initially treated with the cessation of long chain fat and the use of high doses of glucose polymer every 2–3 h. The CK normalised within 48 h using this plan. Mean CK and ALT levels in the 4 years prior to pregnancy, at different stages of pregnancy, and post‐partum are shown in Figure 1. One‐way ANOVA comparison of mean CK values during pregnancy and in the period of 4 years before pregnancy and post‐partum showed statistically significant low CK level (*p* < 0.05) during pregnancy compared with pre‐pregnancy and post‐partum periods.

### 3.4 Delivery

A detailed dietary management plan was devised with the patient and the maternity hospital in advance of the birth. Owing to patient preference, it was decided that delivery would be by caesarean section at 37 weeks gestation, unless a medical need for earlier delivery emerged. However, there was no contra‐indication to vaginal delivery noted.

The mode of anaesthesia for birth was to be decided based on coagulation and transaminase parameters. There was no contraindication noted to oxytocin use peri‐partum nor to non‐steroidal anti‐inflammatories in the post‐partum period. Normal saline was indicated as the resuscitative fluid. Ondansetron was used to manage nausea.

From a dietetic perspective, a reduction in LCT on the day before and the day of delivery was instigated, with a gradual increase back to normal LCT intake post‐partum. An increase in energy was provided via the use of a glucose polymer prior to and after the delivery. Dextrose 15% with appropriate electrolyte supplementation was used during the delivery and immediate recovery period. MCT supplementation was maintained throughout using the patient's usual dietary plan.

An elective caesarean section was performed at 37 weeks gestation under spinal anaesthesia (using bupivacaine 0.5%/glucose 320 mg/4 ml 12 mg, 20 mcg fentanyl and 100 mcg morphine hydrochloride). Cefuroxime 1500 mg IV was administered as antimicrobial prophylaxis. Oxytocin 5 IU was administered intravenously following delivery. In addition, ondansetron 4 mg was administered intra‐operatively. Diclofenac and paracetamol were given per rectum following the procedure for analgesia.

A male infant weighing 3.1 kg was delivered, with Apgar scores of 9 at 1 min and 10 at 5 min. There was meconium Grade 2 liquor present at birth. The caesarean section was uncomplicated, with 400 ml blood loss. Postnatally, the infant remained on the ward with Mum and underwent serial pre‐feed screening for hypoglycaemia. The baby is cared for at home by our patient and her partner with no documented medical issues to date.

## 4 DISCUSSION

There is currently very limited data on the management of pregnancy in patients with LCHADD. The first published information on the management of pregnancy in LCHADD was on a patient with the same genotype (homozygous for G1528C) as our patient.^13^ The patient's blood results remained mostly stable during the pregnancy except during moderate daily life activities, strenuous exercise, or injury. There were no blood pressure control issues documented.

Acute fatty liver of pregnancy (AFLP) is a potentially life‐threatening disorder. It tends to occur in the third trimester or early post‐partum period.^17^ Risk factors for AFLP are primiparity, pre‐eclampsia, male foetus and multiple gestation.^18^ The risk of AFLP in a mother with LCHADD is higher if the mother is a carrier and the foetus is affected rather than if the mother had LCHADD and the foetus was a carrier.^19^ AFLP and HELLP were considered a potential complication in our patient. Apart from deranged liver enzymes, no further deterioration in liver function or evidence of coagulopathy was detected in our patient.

CK levels were strongly correlated with our patient's engagement in daily life activities. Our patient experienced several episodes of metabolic decompensation at different stages of her life requiring hospital admission. This is reflected in CK and ALT levels before pregnancy. During pregnancy, CK levels increased on different occasions up to 3–7 times the upper normal limits during moderate activities such as walking for short distance. However, the magnitude of CK level increases during pregnancy was much lower than pre‐pregnancy and post‐partum. Reductions in CK levels have been reported in in the second and third trimester of pregnancy in VLCAD pregnancies, and have been attributed to fatty acid oxidation by the placenta.^11^, ^12^ Improved CK levels during pregnancy and reduced levels of a number of acylcarnitine derivatives in the third trimester suggest a similar response in LCHADD given that our patient's activity was not significantly reduced during pregnancy.

Although normal vaginal birth is a reasonable approach, caesarean birth was performed in our case because of the potential risk of rapidly deteriorating maternal health, as well as maternal preference. The baby underwent newborn metabolic screening as per national guidelines.

In conclusion, a multi‐disciplinary approach with close medical monitoring and regular dietetic interventions are the cornerstones in the management of LCHADD under challenging physiological conditions such as pregnancy and delivery.

## CONFLICT OF INTEREST

Jenny McNulty received a speaker honorarium from Nutricia Medical and Vitaflo. All authors declare no potential conflict of interest.

## AUTHOR CONTRIBUTIONS

**Loai A Shakerdi:** formal analysis, software, methodology, writing original draft. **Jenny McNulty:** formal analysis, methodology, writing original draft. **Barbara Gillman:** formal analysis, methodology writing original draft. **Claire M. McCarthy:** data curation, methodology, writing original draft. **Jessica Ivory:** data curation, methodology. **Alison Sheerin:** data curation, methodology. **James O'Byrne:** validation, methodology. **Jennifer C. Donnelly:** validation, methodology. **Eileen P. Treacy:** conceptualisation, validation, resources, writing (review and editing).

## Supporting information


**Data S1** Supplementary Tables

## Data Availability

The data included as electronic supplementary material with this paper.

## References

[1] den Boer ME , Wanders RJ , Morris AA , et al. Long‐chain 3‐hydroxyacyl‐CoA dehydrogenase deficiency: clinical presentation and follow‐up of 50 patients. Pediatrics. 2002;109:99‐104. 11773547 10.1542/peds.109.1.99

[2] Spiekerkoetter U , Lindner M , Santer R , et al. Treatment recommendations in long‐chain fatty acid oxidation defects: consensus from a workshop. J Inherit Metab Dis. 2009;32:498‐505. 19452263 10.1007/s10545-009-1126-8

[3] Wallimann T , Wyss M , Brdiczka D , Nicolay K , Eppenberger HM . Intracellular compartmentation, structure and function of creatine kinase isoenzymes in tissues with high and fluctuating energy demands: the ‘phosphocreatine circuit’ for cellular energy homeostasis. Biochem J. 1992;281(Pt 1):21‐40. 1731757 10.1042/bj2810021 PMC1130636

[4] Ventura‐Clapier R , Kuznetsov A , Veksler V , Boehm E , Anflous K . Functional coupling of creatine kinases in muscles: species and tissue specificity. Mol Cell Biochem. 1998;184:231‐247. 9746324

[5] Karall D , Brunner‐Krainz M , Kogelnig K , et al. Clinical outcome, biochemical and therapeutic follow‐up in 14 Austrian patients with long‐chain 3‐Hydroxy acyl CoA dehydrogenase deficiency (LCHADD). Orphanet J Rare Dis. 2015;10:21. 25888220 10.1186/s13023-015-0236-7 PMC4407779

[6] Cervellin G , Comelli I , Lippi G . Rhabdomyolysis: historical background, clinical, diagnostic and therapeutic features. Clin Chem Lab Med. 2010;48:749‐756. 20298139 10.1515/CCLM.2010.151

[7] Gillingham MB , Connor WE , Matern D , et al. Optimal dietary therapy of long‐chain 3‐hydroxyacyl‐CoA dehydrogenase deficiency. Mol Genet Metab. 2003;79:114‐123. 12809642 10.1016/s1096-7192(03)00073-8 PMC2813192

[8] Gillingham M , Van Calcar S , Ney D , et al. Dietary management of long‐chain 3‐hydroxyacyl‐CoA dehydrogenase deficiency (LCHADD). A case report and survey. J Inherit Metab Dis. 1999;22:123‐131. 10234607 10.1023/a:1005437616934 PMC2694044

[9] Herrera E . Lipid metabolism in pregnancy and its consequences in the fetus and newborn. Endocrine. 2002;19:43‐55. 12583601 10.1385/ENDO:19:1:43

[10] Herrera E . Metabolic adaptations in pregnancy and their implications for the availability of substrates to the fetus. Eur J Clin Nutr. 2000;54(Suppl 1):S47‐S51. 10805038 10.1038/sj.ejcn.1600984

[11] Mendez‐Figueroa H , Shchelochkov OA , Shaibani A , Aagaard‐Tillery K , Shinawi MS . Clinical and biochemical improvement of very long‐chain acyl‐CoA dehydrogenase deficiency in pregnancy. J Perinatol. 2010;30:558‐562. 20668464 10.1038/jp.2009.198

[12] Van Calcar SC , Sowa M , Rohr F , et al. Nutrition management guideline for very‐long chain acyl‐CoA dehydrogenase deficiency (VLCAD): an evidence‐ and consensus‐based approach. Mol Genet Metab. 2020;131:23‐37. 33093005 10.1016/j.ymgme.2020.10.001

[13] van Eerd DC , Brusse IA , Adriaens VF , et al. Management of an LCHADD patient during pregnancy and high intensity exercise. J Inherit Metab Dis Rep. 2017;32:95‐100. 10.1007/8904_2016_561 PMC5355378 27334895

[14] Ijlst L , Hoovers JM , Jakobs ME , Wanders RJ . Common missense mutation G1528C in long‐chain 3‐hydroxyacyl‐CoA dehydrogenase deficiency. Characterization and expression of the mutant protein, mutation analysis on genomic DNA and chromosomal localization of the mitochondrial trifunctional protein alpha subunit gene. J Clin Invest. 1996;98(4):1028‐1033. 8770876 10.1172/JCI118863 PMC507519

[15] Gillingham MB , Scott B , Elliott D , et al. Metabolic control during exercise with and without medium‐chain triglycerides (MCT) in children with long‐chain 3‐hydroxy acyl‐CoA dehydrogenase (LCHAD) or trifunctional protein (TFP) deficiency. Mol Genet Metab. 2006;89:58‐63. 16876451 10.1016/j.ymgme.2006.06.004 PMC2706834

[16] Christian P , West KP Jr , Khatry SK , et al. Night blindness of pregnancy in rural Nepal – nutritional and health risks. Int J Epidemiol. 1998;27:231‐237. 9602403 10.1093/ije/27.2.231

[17] Fesenmeier MF , Coppage KH , Lambers DS , Barton JR , Sibai BM . Acute fatty liver of pregnancy in 3 tertiary care centers. Am J Obstet Gynecol. 2005;192:1416‐1419. 15902124 10.1016/j.ajog.2004.12.035

[18] Ko H , Yoshida EM . Acute fatty liver of pregnancy. Can J Gastroenterol. 2006;20:25‐30. 16432556 10.1155/2006/638131 PMC2538964

[19] Treem WR , Rinaldo P , Hale DE , et al. Acute fatty liver of pregnancy and long‐chain 3‐hydroxyacyl‐coenzyme A dehydrogenase deficiency. Hepatology. 1994;19(2):339‐345. 8294091

