# Supplementary material for: Management of pregnancy in a patient with long‐chain 3‐hydroxyacyl CoA dehydrogenase deficiency
Source: JIMD Rep. 2022 Apr 12;63(4):265–70. doi: 10.1002/jmd2.12284 (PMC9259390; doi:10.1002/jmd2.12284)
Supplement: Supplementary file 1 — Data S1 Supplementary Tables [file JMD2-63-265-s001.docx]

## Appendix and supplementary data

Table 2: Diagnostic Acylcarnitine profiles

| [^13^C]-Palmitate loading test; Acylcarnitine profile | | |
| --- | --- | --- |
| nmol/96hr.mg protein | Patient | Controls mean (range) |
| Activity (formed [^13^C]-acetyl carnitine) | 21 | 25 (7-43) |
| Formed [^13^C]-Hydroxypalmitoyl-carnitine (OHC16) | 2.13↑↑ | 0.04 (0-0.1) |
|  | | |
| Overall β-oxidation | | |
| nmol/uur.mg | Patient | Controls mean ± SD (n) |
| [9,10-^3^H] Oleic acid | 1.92↓↓ | 8.23 ±2.06 (22) |
|  | | |
| 3-Hydroxy acyl-CoA Dehydrogenase | | |
|  | Patient | Control in experiment |
| Acetoacetyl-CoA (C4) | 146 | 105 |
| 3-ketohexadecanoyl-CoA (C16) | 23↓↓ | 64 |
| 3-ketohexadecanoyl-CoA/Acetoacetyl-CoA activity ratio | 0.16↓↓ | 61 |
|  | | |
| Long-chain 3-keto acyl-CoA Thiolase | | |
| nmol/min.mg | Patient | Control in experiment |
| 3-ketohexadecanoyl-CoA (C16) | 4.8 | 4.5 |

Table 3: Essential fatty acid (EFA) profile (PUFAs)

|  | September /2015 | December/2020 | July/2021 | NR (umol/L) |
| --- | --- | --- | --- | --- |
| C14:0 Myristic acid | 3.1 | 2.7 | 3.0 | 1.6 – 3.3 |
| C16:0 Palmitic acid | 161.8 | 141.6 | 145.5 | 132 - 190 |
| C18:0 Stearic acid | 119.2 | 115.9 | 116.6 | 91 - 128 |
| C20:0 Arachidic acid | **8.9** | **7.4** | **6.6** | 2.5 – 3.9 |
| C22:0 Behenic cid | 12.8 | **18.1** | **13.9** | 8.1 – 12.9 |
| C24:0 Lignoceric acid | 12.5 | 17.7 | 14.1 | 18.5 – 27.7 |
| C18:3w3 Linolenic acid | 1.0 | 0.7 | 1.0 | 0.5 – 1.3 |
| C20: 5w3 Eicosapentaenoic acid | 3.2 | **0.4** | 2.3 | 1.7 – 7.1 |
| C22:5w3 Docosapentaenoic acid | **20.3** | **17.6** | 12.5 | 7.0 – 14.0 |
| C22:6w3 Docosahexaenoic | 22.4 | 21.0 | 16.7 | 11.2 – 26.6 |
| C14:1w5 Myristoleic acid | **0.1** | **-** | **0.0** | 0.2 – 0.6 |
| C18:2w6 Linoleic acid | 58.3 | 41.3 | 52.8 | 47 - 80 |
| C20:2w6 Eicosadienoic acid | 1.6 | 1.4 | 1.2 | 1.0 – 2.3 |
| C20:3w6 Homogamma-Linolenic acid | 9.8 | **56** | 10.0 | 6.7 – 15.8 |
| C20:4w6 Arachidonic acid | 90.4 | 68.6 | 80.6 | 71 - 104 |
| C22:4w6 Docosatetraenoic acid | 16.6 | 14.3 | 12.4 | 10.5 – 19.3 |
| C22:5w6 Docosapantaenoic acid | 2.8 | 2.3 | 2.0 | 1.2 – 3.6 |
| C16:1w7 Palmitoleic acid | 3.0 | 1.6 | 1.6 | 1.5 – 4.1 |
| C18:1w7 Vaccenic acid | 9.9 | 8.4 | 9.5 | 6.0 – 10.2 |
| C16:1w9 Hypogeic acid | 1.6 | 0.8 | 1.3 | 0.4 – 2.2 |
| C18:1w9 Oleic acid | 83.5 | 65.2 | 79.1 | 61 - 95 |
| C20:3w9 Eicosatrienoic acid | 0.3 | 0.1 | 0.2 | 0.2 – 0.8 |
| C24:1w9 Nervonic acid | 21.5 | 23.6 | 18.8 | 15.9 – 25.7 |
| Total fatty acids | 666.0 | 628.1 | 603.8 | 530 - 762 |

Table 4: Acyl Carnitine Profile during pregnancy and after delivery

|  |  | 19/11/2020 | 04/02/2021 | 27/07/2021 | NR (umol/L) |
| --- | --- | --- | --- | --- | --- |
| Free Carnitine |  | **11.2** | 17.9 | 7.6 | 15.5-46.7 |
| C5DC/C10-OH |  | **0.08** |  |  | 0.0- 0.07 |
| C14-OH- Acylcarnitine |  | 0.01 | 0.00 | 0.02 | 0.0-0.07 |
| Palmitoyl carnitine |  | **0.26** | **0.26** |  | 0.33-1.50 |
| C16-OH- Acylcarnitine |  | **0.10** | 0.06 | 0.15 | 0.0-0.06 |
| Oleyl carnitine |  |  | **0.35** |  | 0.39-1.51 |
| Stearoyl carnitine |  |  | **0.13** |  | 0.21-1.22 |
| C18: 1-OH- Acylcarnitine |  | **0.12** | **0.12** | 0.26 | 0.0-0.07 |
| C18-OH- Acylcarnitine |  | **0.18** | **0.11** | 0.29 | 0.0-0.05 |
| C20:0 Arachidic acid |  | **7.4** |  |  | 2.5 – 3.9 |
| C22:5w3 D0cosapentaenoic acid |  | **17.6** |  |  | 7 – 14 |
| C20: 3W6 Homogamma-Linolenic acid |  | **56** |  |  | 6.7 – 15.8 |
